# Efficacy and Safety of 1 L PEG-ASC Preparation for Colonoscopy in Patients with Inflammatory Bowel Diseases: A Multicenter Observational Study

**DOI:** 10.3390/jcm14145043

**Published:** 2025-07-16

**Authors:** Antonio Facciorusso, Elisa Stasi, Armando Dell’Anna, Mattia Brigida, Eyad Gadour, Aymen Almuhaidb, Badr Al-Bawardy, Marcello Maida, Rodolfo Sacco

**Affiliations:** 1 Gastroenterology Unit, Department of Experimental Medicine, Università del Salento, 73100 Lecce, Italy; elisastasi2@gmail.com (E.S.); armando.dellanna@asl.le.it (A.D.);; 2 Multi-Organ Transplant Centre of Excellence, Liver Transplantation Unit, King Fahad Specialist Hospital, Dammam 32253, Saudi Arabia; eyadgadour@doctors.org.uk; 3 Department of Medicine, Faculty of Medicine, Zamzam University College, Khartoum 11113, Sudan; 4 Department of Medicine, Gastroenterology Division, King Faisal Specialist Hospital and Research Center, Riyadh 11211, Saudi Arabia; draymen86.mu@gmail.com (A.A.); badr.albawardy@yale.edu (B.A.-B.); 5 College of Medicine, Al-Faisal University, Riyadh 11533, Saudi Arabia; 6 Department of Internal Medicine, Section of Digestive Diseases, Yale School of Medicine, New Haven, CT 06510, USA; 7 Department of Medicine and Surgery, University of Enna Kore, 94100 Enna, Italy; marcello.maida@unikore.it; 8 Gastroenterology Unit, Department of Medical and Surgical Sciences, University of Foggia, 73122 Foggia, Italy; rodolfo.sacco@unifg.it

**Keywords:** bowel preparation, IBD, ulcerative colitis, Crohn’s disease, endoscopy

## Abstract

**Background/Objectives**: The effectiveness of 1 L PEG-ASC preparation in inflammatory bowel disease (IBD) patients is still unclear. The aim of this study was to determine the efficacy and safety of 1 L PEG-ASC in a series of IBD patients. **Methods**: Data from a study conducted on a series of 284 patients collected in three centers between 2020 and 2025 were analyzed. The primary outcome was cleansing success whereas success in the right colon, polyp detection, adverse events, and patient reported outcomes were secondary endpoints. **Results**: The mean age was 43.28 ± 12.21 years and 170 (59.8%) patients were male. Out of 141 ulcerative colitis (UC) patients, 45 (32%) presented with a Mayo score of 3. The Mean Simple Endoscopic Score Crohn’s Disease (SES-CD) score in CD patients was 7.2 ± 7.04. Overall and right colon cleansing success were reported in 267 patients (94.2%), of which 134 CD (93.9%) and 133 UC (94.6%) patients (*p* = 0.81). The overall BBPS score was 7.47 ± 1.44, specifically 7.52 ± 1.23 in CD and 7.40 ± 1.62 in UC patients (*p* = 0.76). Overall, 19 patients (6.7%) were diagnosed with polyps with a mean number of polyps per colonoscopy of 0.08 ± 0.03. The preparation was completely taken by 264 (92.9%) patients, and 247 patients (86.9%) declared their willingness to repeat the bowel preparation. Severe nausea was observed in 1 (0.3%) patient and no other severe adverse events were recorded. **Conclusions**: The 1 L PEG-ASC preparation is well-tolerated and safe in IBD patients.

## 1. Introduction

Crohn’s disease (CD) and ulcerative colitis (UC) are potentially disabling diseases and require lifelong management and strict follow-up [1]. Colonoscopy plays a crucial role for the diagnosis, management, and surveillance of inflammatory bowel disease (IBD) patients and proper bowel preparation is well-known to increase the diagnostic performance of the endoscopic procedure.

Specifically, mucosal healing represents a crucial outcome in IBD, because it decreases the recurrences of the disease and the need for surgery [2]; hence, the need for adequate bowel cleansing both for assessment of mucosal healing and for the proper detection of pre-neoplastic lesions during surveillance. Unfortunately, the ingestion of high-volume solutions may impair the tolerability and the compliance of the patients, thus leading to suboptimal cleansing [3]. In fact, the selection of bowel preparation agents for IBD patients warrants special consideration due to their susceptibility to adverse events (e.g., abdominal pain, electrolyte imbalances post-preparation), particularly during active disease, thus leading generally to reduced tolerance in this population.

Recently, a very low-volume 1 L polyethylene glycol plus ascorbate (PEG-ASC) solution (Plenvu^®^; Norgine, Harefield, UK) has been developed and introduced in the clinical practice to improve patients’ compliance to bowel preparation for colonoscopy through the reduction in the total intake of liquids to be consumed. The efficacy of this solution has been shown by several parallel phase-3 randomized controlled trials (RCTs) [4,5,6,7,8,9] and some real-life multicenter studies showing higher effectiveness both in terms of cleansing success and improved adenoma detection rate (ADR) as compared to traditional 2 and 4 L PEG-based preparations [9,10]. As a consequence, this very low-volume preparation could represent the ideal bowel preparation regimen in patients with IBD where repeated endoscopic diagnostic procedures are needed. However, the effectiveness and safety of this preparation in IBD patients has been scarcely studied and evidence supporting its use in this setting is based mainly on a retrospective real-world study [11] and a single-center prospective series [12]. On these premises, the aim of this study was to evaluate the effectiveness and safety of 1 L PEG-ASC preparation in a real-life series of IBD patients collected in three centers.

## 2. Materials and Methods

From a prospectively collected database, the data of IBD patients undergoing colonoscopy with 1 L PEG-ASC preparation in 3 referral Italian endoscopy units were collected between September 2020 and April 2025. All the colonoscopies were performed by experienced endoscopists with specific expertise in IBD endoscopy.

In- and out-patients aged between 18 and 85 years with IBD undergoing screening, surveillance or diagnostic colonoscopy were recruited. All patients underwent an afternoon-morning preparation with 1 L-PEG-ASC (PLENVU, Norgine, Harefield, UK). At the time of colonoscopy scheduling, each patient was provided with a form containing information about the bowel preparation regimen and the instructions for the preparation.

Exclusion criteria were as follows: (1) severe constipation (requiring repeated use of laxatives or manual evacuation), gastrointestinal obstruction or bowel perforation, megacolon; (2) previous gastrointestinal surgery excluding ileoceal resection; (3) glucose-6-phosphate dehydrogenase deficiency or phenylketonuria; (4) hypersensitivity to PEG, ascorbate or sulfates; (5) electrocardiogram changes, heart failure NYHA III/IV or uncontrolled hypertension (systolic blood pressure > 170 mmHg and diastolic pressure > 100 mmHg); (6) severe kidney failure (GFR < 30 mL/min/1.73 m^2^); (7) liver cirrhosis Child Pugh B or C; (8) known severe electrolytic imbalance; (9) pregnant patients or cognitive impairment; (10) indication to colonoscopy for removal of foreign bodies or decompression; (11) drug or alcohol abuse in the last 12 months.

The assessment of disease severity was based on a pre-endoscopic clinical triage using the partial Mayo score for UC (severe disease: partial Mayo score > 7) and the Simple Endoscopic Score for CD (severe disease: SES-CD > 15).

The study received Ethics Committee approval, and the protocol was registered with the number 0522/2020. The study was conducted in accordance with the principles of the Declaration of Helsinki and good clinical practice. Informed consent for participation was obtained from all subjects involved in the study.

### 2.1. Bowel Preparation

All patients followed an afternoon-morning regimen as per current guidelines. The solution was prepared with 500 mL of additional clear fluids after each dose, and additional clear fluids ‘ad libitum’ were permitted up to two hours before the procedure. A low-fiber diet was recommended in the three days preceding the colonoscopy, while on the day before the colonoscopy patients were permitted a light breakfast and lunch. Only clear liquids were permitted at dinner the evening before the colonoscopy.

### 2.2. Outcomes

The primary outcome was cleansing success, defined as a total Boston Bowel Preparation Scale (BBPS) ≥ 6 with a partial BBPS ≥ 2 in each colon segment. Secondary outcomes were the rates of adequate cleansing in the right colon and in the other colon segments (BBPS ≥ 2), patient compliance and patient reported outcomes (including compliance with the diet, proportion of preparation completed, preparation taste, impact of preparation on daily routine, willingness to repeat the bowel preparation), polyps detected during the colonoscopy, preparation-related adverse events (AEs). AE severity was classified according to the ASGE lexicon [13].

### 2.3. Statistical Analysis

Based on a previous RCT assessing the effectiveness of 1 L PEG-ASC6, assuming an expected cleansing success of 97.3% with 1 L PEG-ASC compared to 92.2% with other low volume preparations and considering a drop-out rate of 10%, we planned to enroll 284 patients in our study (with power 90% and probability of type-1 error of 5%).

Continuous variables were expressed as mean ± standard deviation, whereas categoric variables were expressed as frequency and percentage. An independent-samples *t*-test and χ^2^ test were used for comparison of continuous and categorical variables, respectively, between UC and CD patients. All analyses were 2-tailed, and differences were considered significant at a *p* value < 0.05. Statistical analyses were performed using the tableone package in R Statistical Software 3.0.2 (Foundation for Statistical Computing, Vienna, Austria).

## 3. Results

### 3.1. Baseline Characteristics of Patients

Out of 355 patients initially screened for eligibility, 71 were excluded because they did not fulfill the inclusion criteria. Therefore, 284 patients were finally included in the study. The baseline characteristics of the whole study population were reported in Table 1. The mean age was 43.28 ± 12.21 years and 170 (59.8%) patients were male. Comorbidities were registered in 71 (25%) patients. Out of 284 IBD patients included, 143 (50.3%) were CD and 141 (49.7%) were UC patients. Sixty-five (20%) patients had a previous IBD-related surgery. The mean disease duration was 4 ± 3.5 years and disease extension was classified as L1 in 48 patients (34%), L2 in 10 patients (7.1%), and L3 in 85 (58.9%) patients with Crohn’s disease. On the other hand, UC patients were in stage E1, E2, and E3 in 25 (18%), 35 (25%), and 81 (57%) patients, respectively. Most of the patients (76.9%) were under therapy for IBD, mainly with TNFi (40.4%) and mesalazine (22.1%). Out of 141 UC patients, 45 (32%) presented with a Mayo score of 3, 26 (18.4%) were with a Mayo score of 2, 32 (22.6%) with a Mayo score of 1 and 38 (27%) with a Mayo score of 0. The mean SES-CD score in CD patients was 7.2 ± 7.04. The cecal intubation rate was 98%. Sedation was used in all the colonoscopies.

### 3.2. Cleansing Success

**Figure 1:**
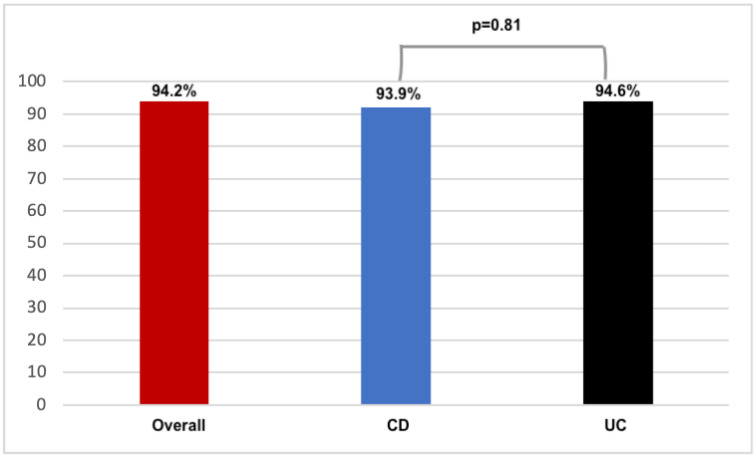
Cleansing success rate.

Effectiveness outcomes are reported in Table 2. Cleansing success was reported in 267 patients (94.2%), of which 134 CD (93.9%) and 133 UC (94.6%) patients (*p* = 0.81; Figure 1). Similar rates were observed in terms of cleansing success in the right colon.

**Figure 2:**
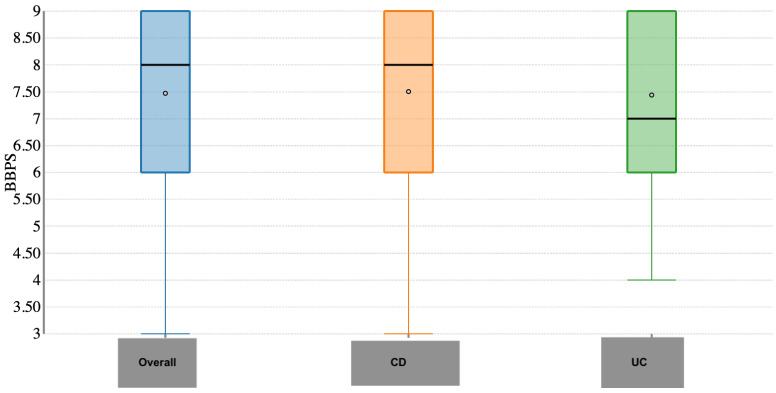
Overall Boston bowel preparation score.

**Figure 3:**
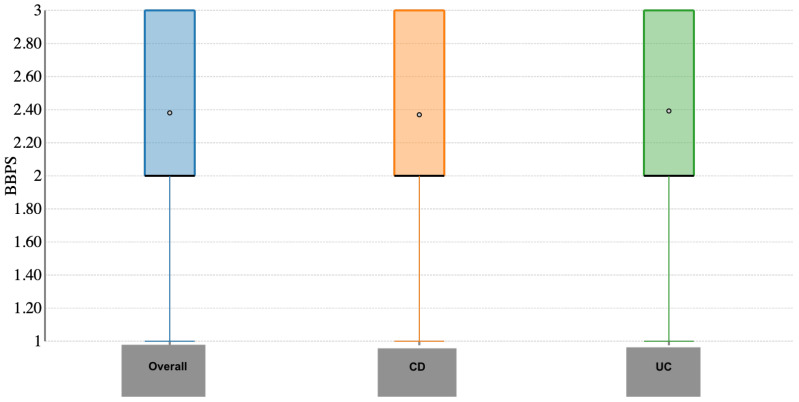
Boston bowel preparation score in the ascending colon.

Rates of adequate cleansing in the transverse and descending colon were 280 (98.5%) and 278 (97.8%), respectively. Again, no difference between CD and UC patients was observed (*p* = 0.97 and *p* = 0.92, respectively). Overall, the BBPS score was 7.47 ± 1.44, specifically 7.52 ± 1.23 in CD and 7.40 ± 1.62 in UC patients (*p* = 0.76; Figure 2). BBPS in the ascending colon was 2.38 ± 0.59, of which 2.36 ± 0.59 in CD and 2.39 ± 0.59 in UC patients (*p* = 0.54; Figure 3). BBPS in the transverse and descending colon was 2.53 ± 0.52 and 2.55 ± 0.53, respectively, with no difference between CD and UC patients (*p* = 0.68 and *p* = 0.23). The cleansing success rate in patients with IBD-related surgery was 54/57 (94.7%) and it was 94% in patients with active disease.

### 3.3. Polyps Detected During Colonoscopy

Overall, 19 patients (6.7%) were diagnosed with polyps with a mean number of polyps per colonoscopy of 0.08 ± 0.03. Most of the polyps were between 6 and 10 mm (45.8%), were classified as Paris Is (54.1%), and were resected with a cold snare technique (37.5%). Out of 14 polyps retrieved and sent to histological examination, 6 were adenomas, 7 were hyperplastic, and 1 was a pseudopolyp.

### 3.4. Compliance to Bowel Preparation and Patients’ Reported Outcomes

A detailed list of patients’ reported outcomes was reported in Table 3.

Compliance with the diet was registered in 275 (96.8%) patients. The preparation was completely taken by 264 (92.9%) patients whereas only 1 subject (0.4%) took less than 50% of the preparation.

The preparation taste was rated according to validated scales [14,15] and reported as very bad in 48 cases (16.9%), mediocre in 85 cases (29.9%), and decent in 71 cases (25%). On the other hand, 59 (20.7%) and 21 patients (7.5%) rated it as good and very good, respectively.

The impact of bowel preparation on the daily routine was considered mainly null (46.7%) or only low (36.2%). Most of the patients (86.9%) declared to be willing to repeat the bowel preparation if needed.

### 3.5. Adverse Events

Adverse events are reported in Table 4. Severe nausea was observed in 1 (0.3%) patient and no other severe AEs were reported. Mild abdominal bloating was experienced in 32 (11.2%) patients. Abdominal pain was classified as moderate and mild in 1 (0.3%) and 17 (5.9%) patients, respectively.

No other preparation-related symptoms were reported in our series.

## 4. Discussion

Adequate bowel preparation is crucial in IBD patients because it enables us to perform enhanced imaging and chromoendoscopy, the diagnostic gold standard to detect IBD-associated dysplasia/colorectal cancer [16], and to assess properly endoscopic IBD activity. Since previous studies demonstrated that IBD patients reported bowel preparation as the most important reason not to comply with surveillance recommendations [17], improving this aspect is of paramount importance to ameliorate the management of these patients. Although low-volume preparations were found to be more effective and better tolerated as compared with other high-volume solutions both in general settings [18,19] and specifically in IBD patients [20,21], the definition of the optimal bowel preparation regimen in IBD subjects is still unclear.

A recent very low-volume preparation, namely 1 L PEG-ASC, showed interesting results in IBD patients due to its high tolerability and effectiveness. However, there are still scarce data supporting its use in this setting mainly derived from retrospective real-world data [11,12]; on the other hand, a recent small RCT comparing different low-volume solutions showed no difference between 1 L PEG, 2L PEG, and sodium picosulfate that in turn was better tolerated [22].

Therefore, we decided to conduct a real-life study assessing the efficacy and safety of 1 L PEG-ASC in a cohort of IBD patients; particularly, our study was the first reporting of several patient-reported outcomes related to the tested preparation. Overall cleansing success and adequate bowel preparation in the right colon were reported in 267 patients (94.2%). Slightly higher rates of adequate cleansing were observed in the other colon segments. No difference in terms of efficacy between CD and UC patients was observed.

Our results were in keeping with previous retrospective studies evaluating 1 L PEG-ASC in IBD patients [11,12]. Of note, our prospective series did not include patients with severe constipation and who did not perform a split preparation, two characteristics that resulted as significant predictors of cleansing unsuccess in the study by Maida et al. [11].

The high effectiveness of 1 L PEG-ASC in IBD patients might be due to its tolerability and the low volume of the solution that the patient should take. In fact, patient compliance was very high in our study and the preparation was completed taken in the vast majority of cases, thus further increasing the adherence to the regimen. Only a small proportion of patients (17.5%) rated the preparation taste as very bad, in keeping with the current literature concerning 1 L PEG-ASC [23]; similarly, patients’ experience with this preparation regimen was considered positive as in previous real-life studies [23,24]. The high scores observed in all the patient-reported outcomes in our series showed that the tolerability of 1 L PEG-ASC in IBD patients is at least as high as in the aforementioned studies conducted in the general population.

Therefore, our data contribute to a growing body of evidence showing that 1 L PEG -ASC delivers high effectiveness and good tolerability in both the general population and IBD patients.

Overall, the investigated solution presented a favorable safety profile. Only 1 severe AE was reported, specifically a case of severe nausea, whereas abdominal bloating and pain were mainly mild and none of these AEs led to the interruption of the preparation or caused hospitalization. As observed by Maida et al. [11], a low incidence of vomit was observed whereas the high incidence of abdominal pain represented the main safety concern with this preparation [11]. This might be probably secondary to the presence of the pre-existing IBD, which may affect the presence of abdominal symptoms. To the best of our knowledge, this is the largest real-world cohort study assessing the effectiveness, tolerability, and patient-reported outcomes with 1 L PEG-ASC in patients with IBD.

However, there are some limitations to our study. First of all, the lack of a comparison arm with a randomized design of the study did not enable us to infer on the comparative efficacy of this regimen with other low-volume solutions in IBD patients. Secondly, the absence of blinding could have influenced particularly patient-reported outcomes which are highly subjective. Third, the relatively limited sample size prevented a logistic regression analysis of predictors of cleansing success. Finally, the exclusion of patients with severe constipation or non-split preparations may overestimate cleansing success.

## 5. Conclusions

This study supports the effectiveness and safety of 1 L PEG-ASC in IBD patients, mainly as a consequence of the high tolerability and patient compliance to this regimen. Further RCTs are needed to confirm these results and to compare this solution to other low-volume preparation regimens.

## Figures and Tables

**Table 1:** Baseline characteristics of the study population.

Variable	284 Patients
Age	43.28 ± 12.21
Sex male	170 (59.8%)
Comorbidities	71 (25%)
Disease Crohn’s Ulcerative Colitis	143 (50.3%) 141 (49.7%)
Disease duration	4 ys ± 3.5
Disease extension Crohn’s disease L1 L2 L3	48 (34%) 10 (7.1%) 85 (58.9%)
Disease extension Ulcerative Colitis E1 E2 E3	25 (18%) 35 (25%) 81 (57%)
Previous IBD-related surgery	57 (20%)
Concomitant therapy Steroid Mesalazine TNFi Anti-integrin Other None	17 (6%) 63 (22.1%) 115 (40.4%) 9 (3.1%) 15 (5.3%) 65 (23.1%)
Mayo score 0 1 2 3	38 (27%) 32 (22.6%) 26 (18.4%) 45 (32%)
SES-CD	7.2 ± 7.04

**Table 2:** Cleansing success.

	Overall (284 pts)	CD (143 pts)	UC (141 pts)	*p* Value
Adequate cleansing	267 (94.2%)	134 (93.9%)	133 (94.6%)	0.81
Adequate cleansing ascending colon	267 (94.2%)	134 (93.9%)	133 (94.6%)	0.81
Adequate cleansing transverse colon	280 (98.5%)	140 (97.9%)	140 (99.2%)	0.97
Adequate cleansing descending colon	278 (97.8%)	140 (97.9%)	138 (97.8%)	0.92
Overall BBPS	7.47 ± 1.44	7.52 ± 1.23	7.40 ± 1.62	0.76
BBPS ascending colon	2.38 ± 0.59	2.36 ± 0.59	2.39 ± 0.59	0.54
BBPS transverse colon	2.53 ± 0.52	2.51 ± 0.76	2.54 ± 0.43	0.68
BBPS descending colon	2.55 ± 0.53	2.58 ± 0.53	2.49 ± 0.61	0.23

**Table 3:** Adherence of the patient to the bowel preparation regimen.

Variable	284 Patients
Compliance with the diet	275 (96.8%)
Proportion of preparation completed 100% >75% 50–75% <50%	264 (92.9%) 17 (6%) 2 (0.7%) 1 (0.4%)
Preparation taste Very bad Mediocre Decent Good Very good	48 (16.9%) 85 (29.9%) 71 (25%) 59 (20.7%) 21 (7.5%)
Impact on daily routine High Moderate Low None	7 (2.4%) 42 (14.7%) 103 (36.2%) 132 (46.7%)
Willingness to repeat the bowel preparation	247 (86.9%)

**Table 4:** Adverse events.

Variable	284 pts
Nausea Severe Moderate Mild No	1 (0.3%) 3 (1%) 58 (20.4%) 222 (78.3%)
Abdominal bloating Severe Moderate Mild No	0 (0%) 0 (0%) 32 (11.2%) 252 (88.8%)
Abdominal pain Severe Moderate Mild No	0 (0%) 1 (0.3%) 17 (5.9%) 266 (93.8%)

## Data Availability

Data available upon request.

## Abbreviations

1 L	One liter
PEG-ASC	Polyethylene glycol plus ascorbate
IBD	Inflammatory bowel disease
CD	Crohn’s disease
UC	Ulcerative colitis
BBPS	Boston Bowel Preparation Scale
SES-CD	Simple Endoscopic Score for Crohn’s Disease
AE	Adverse event
RCT	Randomized controlled trial
ADR	Adenoma detection rate
GFR	Glomerular filtration rate
ASGE	American Society for Gastrointestinal Endoscopy
TNFi	Tumor necrosis factor inhibitor
